# Draft Genome Sequences of Two Acinetobacter soli Clinical Isolates from a Tertiary Hospital in Terengganu, Malaysia

**DOI:** 10.1128/mra.00082-22

**Published:** 2022-04-04

**Authors:** Farahiyah Mohd. Rani, Nor Iza A. Rahman, Salwani Ismail, David W. Cleary, Stuart C. Clarke, Chew Chieng Yeo

**Affiliations:** a Centre for Research in Infectious Diseases and Biotechnology, Faculty of Medicine, Universiti Sultan Zainal Abidin, Kuala Terengganu, Malaysia; b Faculty of Medicine and Institute for Life Sciences, University of Southampton, Southampton, United Kingdom; c NIHR Southampton Biomedical Research Centre, University of Southampton, Southampton, United Kingdom; d Global Health Research Institute, University of Southampton, Southampton, United Kingdom; e Institute for Research, Development, and Innovation, International Medical University, Kuala Lumpur, Malaysia; University of Maryland School of Medicine

## Abstract

We report the draft genome sequences of Acinetobacter soli AC1511 and AC15148, which were isolated from a tertiary hospital in Terengganu, Malaysia, in 2015. AC1511 was assembled into 43 contigs with a total genome size of 3,320,693 bp, whereas AC15148 was 3,260,687 bp over 47 contigs.

## ANNOUNCEMENT

Bacteria of the genus Acinetobacter are opportunistic pathogens, and although A. baumannii is the most clinically important species due to its multidrug-resistant characteristics, other non-*baumannii* species, such as A. nosocomialis, A. pittii, and A. soli, are known etiologic agents of nosocomial infections (1). Here, we report the draft genome sequences of two *A. soli* isolates, AC1511 and AC15148, from Hospital Sultanah Nur Zahirah (Terengganu, Malaysia). Ethical approval for the collection of isolates was obtained from the Medical Research and Ethics Committee review board of the Malaysian Ministry of Health (protocol number NMRR-14-1650-23625 [IIR]). AC1511 was isolated from the sputum of a 59-year-old female patient with community-acquired pneumonia in 2015, whereas AC15148 was isolated from the blood of a 3-year-old boy with acute tonsillopharyngitis in 2018. AC1511 and AC15148 were identified as *A. soli* by PCR-amplification and sequencing of the *rpoB* gene (2, 3). Antimicrobial susceptibility was determined using CLSI breakpoints (4) by obtaining the MIC values using Etest strips for carbapenems and cephalosporins and broth microdilution for polymyxins, whereas disc diffusion was used for other antimicrobials. Both *A. soli* AC1511 and *A. soli* AC15148 were susceptible to all antibiotics tested—the carbapenem class (i.e., imipenem, meropenem, and doripenem), cephalosporins (cefotaxime, ceftriaxone, ceftazidime, and cefepime), β-lactam/β-lactamase inhibitor combinations (piperacillin/tazobactam and ampicillin/sulbactam), aminoglycosides (amikacin, gentamicin, and tobramycin), trimethoprim/sulfamethoxazole, fluoroquinolones (ciprofloxacin and levofloxacin), tetracyclines (tetracycline, doxycycline, and minocycline), and polymyxins (polymyxin B and colistin). Genomic DNA was isolated using the Geneaid Presto mini-genomic DNA (gDNA) bacterial kit from a 5-mL overnight culture grown at 37°C in Luria broth. The Nextera XT DNA library preparation kit (Illumina) was used to prepare genomic DNA libraries, which were sequenced on the Illumina HiSeq platform (2 × 150-bp paired-end reads) by a commercial sequencing provider (Novogene) with quality inspection carried out using FastQC v0.11.8 and MultiQC v1.7 (5). The draft genome sequences were assembled using SPAdes v3.11.1 (6). For all software, default parameters were used except where otherwise noted. The genome of *A. soli* AC1511 was assembled into 43 contigs (*N*_50_ value of 246,341 bp, GC content of 43.1%, genome coverage of 150×) with a total genome size of 3,320,693 bp. The *A. soli* AC15148 genome size was 3,260,687 bp and was assembled into 47 contigs with an *N*_50_ value of 173,816 bp, GC content of 42.7%, and genome coverage of 150×. In comparison with the reference *A. soli* strain, GFJ2 (GenBank version number GCF_001953195.1), the genome of AC1511 showed an average nucleotide identity (ANI) of 98.47% using the BLAST algorithm (ANIb) and 98.7% using the MUMmer alignment tool (ANIm) at JSpeciesWS (7). AC15148 gave an ANIb value of 98.43% and an ANIm value of 98.69% compared with *A. soli* GFJ2, thus validating the identities of AC1511 and AC15148 as *A. soli*. Genome annotation was performed using the Prokaryotic Genome Annotation Pipeline v5.1 during sequence submission to NCBI (8). No resistance genes were detected from the genomes of AC1511 and AC15148 using ResFinder (9) and the Comprehensive Antimicrobial Resistance Database (CARD) (10), which confirmed their observed susceptible phenotypes.

### Data availability.

The raw reads used for the draft genome sequence assemblies were deposited in the Sequence Read Archive under accession numbers SRR18110793 (for Acinetobacter soli AC1511) and SRR18120237 (for *A. soli* AC15148). The draft genome sequences of the strains obtained in this study were deposited under BioProject number PRJNA576555 with the assembled genomes under the accession numbers JAGFOS000000000 (*A. soli* AC1511) and JAGFOR000000000 (*A. soli* AC15148).
